# Numerical Study of the Thermal Performance of a Mems Pressure Sensor with Self-Calibration Capabilities

**DOI:** 10.3390/s22103828

**Published:** 2022-05-18

**Authors:** Albrey de Clerck, Yuhong Kang, Ridge Sibold, Scott Mouring, Hang Ruan, Wing Ng

**Affiliations:** 1 Department of Mechanical Engineering, Virginia Polytechnic Institute and State University, Blacksburg, VA 24061, USA; adeclerck@nanosonic.com (A.d.C.); sridge94@vt.edu (R.S.); smouring@vt.edu (S.M.); 2 NanoSonic, Inc., Pembroke, VA 24136, USA; ekang@nanosonic.com

**Keywords:** self-calibrating, intelligent, MEMS, pressure sensor

## Abstract

Recent industry trends toward more complex and interconnected systems have increased the demand for more reliable pressure sensors. By integrating a microactuator with a pressure sensor, the sensor can self-calibrate, eliminating the complexities and costs associated with traditional sensor calibration methods to ensure reliability. The present work is focused on furthering understanding and improving the thermal performance of a thermopneumatic actuated self-calibrating pressure sensor. A transient numerical model was developed in ANSYS and was calibrated using experimental testing data. The numerical model provided insights into the sensor’s performance not previously observed in experimental testing. Furthermore, the model was utilized for two design studies. First, it was found that a substrate with low thermal conductivity and high thermal diffusivity is ideal for both the sensor’s efficiency and a faster transient response time. The second design study showed that decreasing the size of the sealed reference cavity lowers power consumption and transient response time. The study also showed that reducing the cavity base dimension has a greater effect on lowering power consumption and response time. Overall, the present work increases understanding of the self-calibrating pressure sensor and provides insight into potential design improvements, moving closer to optimized self-calibrating pressure sensors.

## 1. Introduction

Most if not all engineering applications rely heavily on sensors to measure the state of a system or process and provide information to computers, operators, or engineers. Pressure sensors are arguably one of the most used sensors and can be found in nearly all applications from lawn irrigation systems to complex aerospace systems, yet pressure sensor technology is relatively underdeveloped in terms of intelligent sensor capabilities. As we progress further into the Information Age, reliance on accurate pressure sensor data is increasing as engineers develop more complex systems. Recent industry trends toward autonomous systems, the Internet of Things (IoT), and embedded systems have further incentivized the development of better performing, more reliable, and smarter pressure sensors.

A variety of pressure sensors exist, but piezoresistive pressure sensors account for a large portion of the sensors market due to their simplicity and favorable performance. A typical piezoresistive pressure sensor consists of four silicon piezoresistors rigidly bonded to a pressure-sensitive membrane. When pressure is applied to the membrane, stress induced in the resistors results in an electrical resistance change via the piezoresistive effect shown in Equation (1):

(1) $$\frac{\Delta R}{R}=\pi_{l}\sigma_{l}+\pi_{t}\sigma_{t}$$

The nomenclatures of all the expressions can be found in Nomenclature.

Configuring the resistors in a Wheatstone bridge, the resistance change is converted to a voltage signal corresponding to the applied pressure. Even though the sensor’s operating principle is simple, much work has been performed to characterize and improve piezoresistive microelectromechanical system (MEMS) pressure sensor design [1]. Some of the work includes sensitivity and linearity optimization via structural design [2], temperature effects and compensation techniques [3], sensor packaging [4,5], and methods to optimize sensor signal-to-noise ratio [6]. However, like all pressure sensors, mechanical wear in piezoresistive sensors still results in failure, hysteresis, signal drift, and nonlinearity errors, culminating in degradation of sensor reliability and accuracy.

The best way to check the functionality of pressure sensors and eliminate the aforementioned errors is by comparing the sensor’s output to a well-defined, standard pressure. This process, known as calibration, produces a curve that quantifies the relationship between the sensor output signal and the input pressure. Traditional calibration procedures account for a significant portion of the manufacturing and maintenance costs of pressure sensors. The costs arise as sensor calibration must be performed regularly over the life of the sensor to ensure accuracy and requires time, money, trained personnel, and specialized equipment.

Advances in MEMS, NEMS (nano-electromechanical system), materials, and cleanroom processes have provided a means for developing self-calibrating pressure sensors, mitigating the drawback of traditional calibration procedures. By incorporating a microactuator and sensor into one small device, the actuator can be used to calibrate the sensor, replacing the need for an external standard as required by traditional calibration procedures. Concepts and methods for various self-calibrating sensors have been developed [7], but this new technology has yet to be fully executed and commercially produced.

Self-calibrating pressure sensors, along with appropriate software algorithms, can ensure data accuracy without the drawbacks and costs of traditional calibration procedures. The need for direct access of the sensor is eliminated, and a simple calibration command initiates and performs the self-calibration sequence. Furthermore, uninstalling the sensor is not required, reducing downtime and costs. Lastly, by minimizing the time required for self-calibration, the functionality and accuracy of the sensor can be checked often, ensuring data quality for time-sensitive and mission-critical applications, such as flight.

The many advantages of self-calibrating pressure sensors are accompanied by two challenges. First, the current self-calibrating pressure range is limited. Sensor calibration standards require that sensors be calibrated over their full sensing range, presenting a challenge as microactuators are limited to small pressures, thus limiting the achievable self-calibration range. The second challenge in developing a self-calibrating pressure sensor is the accuracy requirement. Since the accuracy of the sensor depends directly on the self-calibration, the microactuator needs to be well characterized and precisely controllable to ensure accurate and repeatable calibration curves.

Only two self-calibrating pressure sensors have been found in literature, possibly due to the challenging nature of developing a system capable of performing as previously discussed. Yameogo et al. reported a wireless self-calibrating pressure sensor for biomedical applications [8]. Used to measure intracranial pressure, the sensor is of a piezoresistive type with a pressure range of 0–150 mmHg (~2.9 psi). An electrostatic actuator is used to achieve self-calibration by applying a voltage between two electrodes separated by an air gap. When actuated, the pressure-sensitive membrane deflects, resulting in a sensor output signal change. However, using an actuator other than pressure for a pressure sensor is not ideal as it will be unable to detect potential defects such as a leak in the reference cavity [9]. The second self-calibrating pressure sensor is found in the literature from previous work by the current authors [10]. A microheater and thermistor were integrated into the sealed reference chamber of the sensor, forming a monitorable thermopneumatic actuator, commonly used for microvalves and pumps [11,12]. Combining a thermopneumatic actuator and a piezoresistive sensor has been accomplished before [13,14], but the addition of the cavity thermistor allows for self-calibration. To self-calibrate, the heater is turned on, the gas in the cavity heats up and expands, and the internal pressure acts on the pressure sensor membrane, producing a signal. Using the cavity thermistor, the temperature in the cavity is measured, and a state equation such as the Ideal Gas law is used to calculate the pressure. Knowing the pressure from the actuator and the sensor output, standard calibration procedures can be applied. The previous work presents the operating principle and conceptual design of the sensor. Furthermore, a prototype sensor was built and tested.

Predictive and consistent thermal performance is essential to the sensor’s self-calibrating capabilities. However, current thermal physics understanding is limited to simple analysis and experimental testing. For this reason, the present work uses numerical modeling, validated by experimental data, to provide greater insight into the transient thermal performance of self-calibrating pressure sensors. Furthermore, the numerical model aids in assessing potential design improvements. The numerical model development, calibration with experimental data, numerical results, and sensor improvements inspired by the results are presented in the following sections.

## 2. Model Development

This section starts with a brief overview of the self-calibrating sensor specifications and its principle. Next, the governing physics and model implementation in ANSYS are described. Lastly, the experimental testing, the model calibration procedure, and the results are presented.

### 2.1. Sensor Description

**Figure 1:**
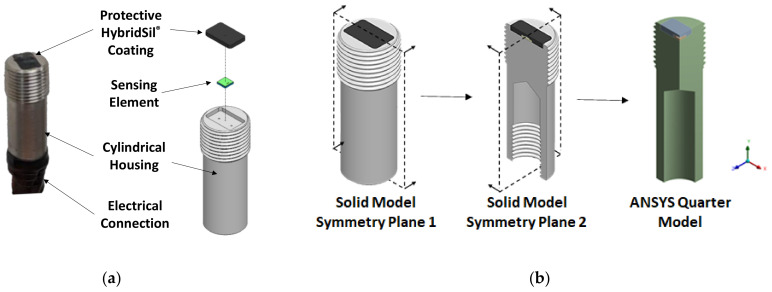
(**a**) Picture (**left**) and CAD model (**right**) of the self-calibrating pressure sensor showing the four main components and (**b**) geometry of the self-calibrating pressure sensor as implemented in ANSYS.

The self-calibrating, piezoresistive sensor studied in this work consists of four main components, as shown in Figure 1a. To ensure linearity, the current pressure range is limited to 35.5 kPa (~5 psi), and the thermopneumatic actuator is limited to a heater voltage of 5 V, corresponding to a heater power of 0.54 W.

**Figure 2:**
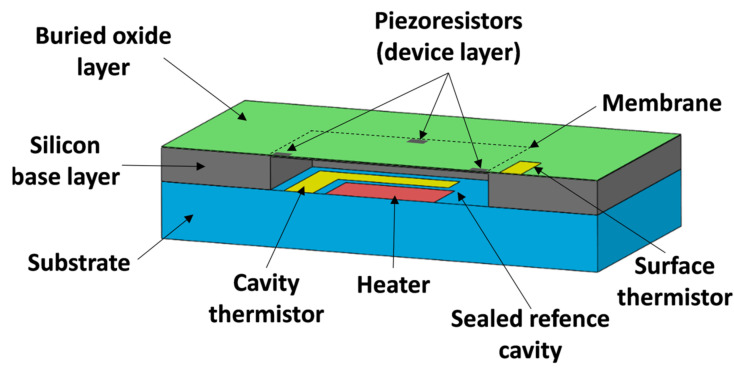
Cross-section view of the self-calibrating pressure sensor sensing element.

The sensing element has a square base measuring 4 mm by 4 mm with a total height of 805μm and is the main component of interest. A close-up, cross-sectional view of the sensing element with all main components is shown in Figure 2 below.

The device layer, the buried oxide (BOX) layer, and the silicon base layer are manufactured from a silicon on insulator (SOI) wafer, while the gold heater and cavity thermistor are manufactured on the substrate. By bonding the substrate to the diced SOI wafer, the sealed reference cavity is created with the enclosed heater and thermistor forming the monitorable thermopneumatic actuator. The surface thermistor on top of the sensing element is used to evaluate the thermal effects on the device layer while acting as a temperature sensor as well.

### 2.2. Self-Calibration Principle

The self-calibrating pressure sensor’s operating principle is derived from the sensing element configuration. As the reference cavity is sealed during the manufacturing process, the sensor measures the pressure difference between the applied pressure and the reference cavity pressure. This pressure differential measurement across the sensing element membrane (∆*P*) is described by Equation (2) below.

(2) $$\Delta P=P_{applied}-P_{reference}$$

**Figure 3:**
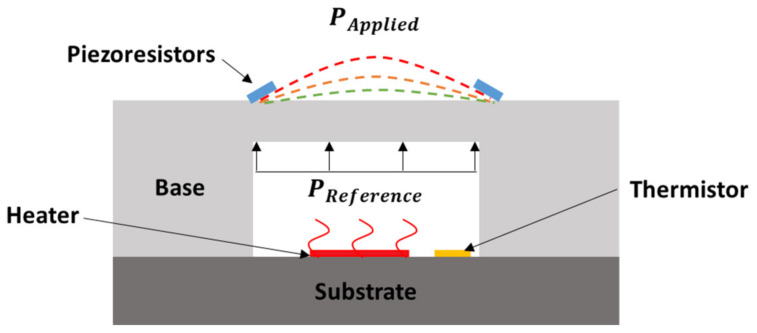
Simplified demonstration of the self-calibrating pressure sensor procedure where varying heater power is used to change the sealed reference cavity pressure, resulting in different sensor responses and calibration points (green, orange, and red dashed lines).

When ∆*P* is positive, the membrane deflects downward into the cavity, and when ∆*P* is negative, the membrane deflects upward. Previous works showed that the change in volume due to membrane deflection is negligible and that the sensor’s response is symmetric whether the membrane deflects downward or upward. Thus, by knowing and controlling the pressure in the sealed reference cavity (*P_reference_*) along with sensor knowledge and the temperature measurements from the two thermistors, the applied pressure (*P_applied_*) can be determined. This process can then be repeated for several different cavity reference pressures (*P_reference_*), resulting in a calibration curve (Figure 3). 

### 2.3. Governing Physics

To model the thermal performance, a good understanding of the governing physics and assumptions is required. Previous work has illustrated that radiation heat transfer is negligible at the anticipated temperature; therefore, this study only focuses on convection and conduction effects. 

Two forms of convective heat transfer can occur in the air-filled reference cavity: forced convection, where a fluidic medium is forced across a surface to increase heat transfer, and buoyancy-induced convection, which relies on temperature-induced density gradients to govern fluid motion. Forced convection was assumed negligible as the volume of air displaced in the cavity (due to the displacing membrane) is less than 3% when compared to the total volume, resulting in minimal bulk fluid motion. 

For buoyancy-induced convection, two criteria must be satisfied. First, an unstable temperature gradient must exist in the presence of a body force (gravity). For the present work, this is true as the heater is located at the bottom of the cavity, resulting in higher temperatures at the bottom as compared to the top of the cavity. In addition to having an unstable temperature gradient, buoyancy forces in the fluid must be greater than the viscous forces. A nondimensional number used for quantifying the comparison of buoyancy forces to viscous forces is the Grashof number (Equation (3)).

(3) $$Gr=\frac{g\beta (T_{1}-T_{2})L^{3}}{v^{2}}$$

Large Grashof numbers (*Gr* >> 1) indicate that buoyancy forces are sufficient to overcome the viscous forces in the fluid, resulting in bulk fluid motion, whereas small *Gr* numbers (*Gr* << 1) indicate that viscous forces dominate; therefore, no bulk fluid motion occurs. Due to the *L^3^* term in the numerator of Equation (3), buoyancy-induced convection in MEMS and NEMS devices can often be neglected [15,16,17]. Similarly, the Gr number for the present work is 0.13, indicating buoyancy-induced convection in the cavity is insignificant.

With no radiation or convection, the sensor’s thermal performance is governed by heat conduction in both the solid and fluid domains. Derived from the energy equation and Fourier’s law, the governing heat conduction equation is shown in Equation (4):

(4) $$\rho C_{p}\left[\frac{\partial T}{\partial t}+U\cdot \vec{\nabla }T\right]=\vec{\nabla }\cdot \left(k\vec{\nabla }T\right)+q^{\prime \prime \prime },$$

where $\vec{\nabla }$ is the gradient operator. When dealing with small length scales, it is important to check the validity of Equation (4) as the thermal conductivity (*k*) could vary from the bulk values. For the present work, it was determined that the length scales are sufficiently larger than the mean free path of the materials such that the bulk thermal conductivity values stand.

For this study, rather than modeling the joule heating effect for the heater, the heater is treated as an input heat flux over the area on which the heater resides, eliminating the volumetric heating term in Equation (4). Furthermore, the velocity term in the governing equation is eliminated due to stationary material, and lastly, constant material properties were assumed. To reduce the number of parameters, thermal diffusivity, the ratio of thermal energy conducting through a material relative to the energy stored in a material, is utilized and defined in Equation (5).

(5) $$\alpha =\frac{k}{\rho C_{p}}$$

The resulting linear, first-order in time and second-order in space governing partial differential equation (PDE) is shown below in Equation (6).

(6) $$\frac{\partial T}{\partial t}=\alpha \nabla^{2}T$$

Along with an initial condition and two boundary conditions, the above equation for the three-dimensional sensor was solved using the finite-element method in ANSYS R19.2.

### 2.4. Numerical Model Setup

#### 2.4.1. Geometry

For modeling purposes, the geometry of the sensor shown in Figure 1 and Figure 2 was simplified. The internal threads, chamfers, and wire pass-through holes of the housing were neglected. Due to the heater, cavity thermistor, surface thermistor, and device layer being on the order of 100 nm thin, their geometries were also neglected; rather, boundary conditions and probe surfaces were used instead. Lastly, as the sensor’s geometry, materials, and loads are symmetric, two symmetry planes were used to quarter the sensor, reducing computational resources and time. The geometry of the sensor as implemented in ANSYS is shown in Figure 1b.

#### 2.4.2. Material Properties

For all work presented, the thermal diffusivities of silicon, silicon dioxide, and air were taken, as shown in Table 1. Due to limited information regarding the exact properties of the glass, stainless steel, and NanoSonic’s HybridSil^®^, the properties in Table 1 were initially assumed, knowing experimental data will be used to calibrate the model by varying these properties, as discussed later. The extended material properties were documented by De Clerck [18].

#### 2.4.3. Mesh

Each part was meshed independently to ensure the mesh was adequate across varying length scales, and perfect thermal contact was assumed between all parts. A three-dimensional tetrahedral thermal element (SOLID87) was used for its ability to mesh intricate geometric features while accurately representing the physics. Each element consists of 10 nodes, with one degree of freedom per node, temperature. The selected element allows for either a convective or heat flux load on the element surface, making it appropriate for this study. 

The elements in each part were assigned a maximum allowable size, and a grid-independence study was performed to ensure the solution was not affected by the mesh. Starting with a coarse mesh, the element size for each part was halved for each subsequent case, and the maximum temperature of each part was monitored. The solution was considered grid-independent when the percent change in maximum temperature of each part was less than 1% compared to the previous coarser mesh density.

**Figure 4:**
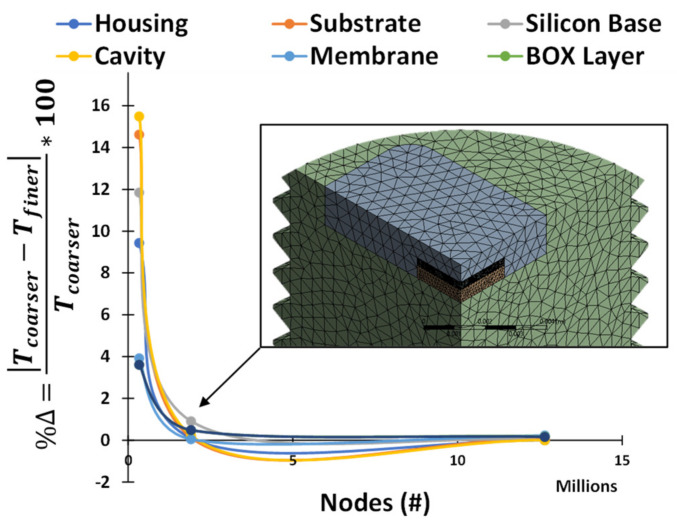
Grid-independence study results with the final selected mesh.

As shown in Figure 4, after the second mesh refinement, the percent change was less than 1% for all parts. A third refinement confined the results with less than 0.5% percent change, but due to the increased computational time, the mesh from the second refinement was selected. The selected mesh consisted of approximately 1.9 million nodes and 1.2 million elements. All the mesh statistics and data can be found in [18].

#### 2.4.4. Initial Condition and Boundary Conditions

The uniform initial temperature for the transient model and the environmental temperature were set to 22.7 °C, matching experimental conditions. Like the material properties, a convective boundary with a convection coefficient (h) of 10 W/(m^2^ °C) was initially assumed for the top and outside surfaces of the sensor in contact with air, knowing experimental data would later be used to calibrate the model. Symmetry boundary conditions were assigned to each of the cut planes forming the quarter sensor. The heater was modeled as a uniform step input heat flux over the area where the heater is located. All other surfaces were assumed to be perfectly insulated. Refer to reference [18] for an illustration of the boundary conditions implemented in ANSYS.

#### 2.4.5. Solution Method and Verification

The model was solved using the Quasilinear Thermal Transient Solution method in ANSYS. Automatic time stepping was enabled to ensure sufficient time resolution over the whole simulated time while minimizing computational resources. A Distributed Sparse Direct equation solver was used to solve the system of linear equations on a 14 core, Intel Core i9-7940X 3.1–4.3 GHz workstation with 128 GB of RAM.

To verify the model setup and solution method, an analytical analysis was conducted and compared to the numerical results. The long cylindrical shape of the sensor housing, with a low Biot number in the radial direction, enables the use of the transient fin equation [19] to verify the numerical solution. The numerical model and fin equation results agree well, verifying the model setup and solution method. Further details regarding model verification can be found in [18].

### 2.5. Numerical Model Calibration

The model uncertainty from the assumed material properties and convection boundary condition was reduced using experimental testing data to calibrate the model. The testing, model calibration procedure, and results are described in the following sections.

#### 2.5.1. Test Setup and Procedure

The prototype self-calibrating sensor was mounted on a test stand using a three-prong extension clamp. A FLIR A325sc Infrared (IR) camera was used to collect temperature data on the outer housing surface, painted with ultra-flat black paint (emissivity of 0.97 at wavelengths of 5 μm) to ensure accurate surface temperature measurements. The heater voltage, thermistors, and pressure signals were recorded at a sampling rate of 25 kHz using a PicoScope 4824 oscilloscope. The heater voltage was also captured on a NI USB-6210 Data Acquisition (DAQ) System and was used to temporally sync the IR data with the oscilloscope data.

The testing procedure started by setting a trigger on the oscilloscope and starting the DAQ and IR camera recordings. When the heater voltage was turned on, the oscilloscope triggered recording. After approximately 15 min, the heater was turned off, and the sensor was left to cool down before all recording was stopped. Starting at a heater voltage of zero, as a baseline, the voltage was increased by 1 V increments up to 5 V and then back down to 1 V to ensure repeatability. Later, the data were postprocessed in MATLAB. Previously obtained NIST traceable calibration curves were used to convert voltages into pressure, temperature, and heater power signals [10]. Following the voltage to signal conversion, a moving mean filter was employed to eliminate high-frequency EMI, as well as other potential white noise sources.

#### 2.5.2. Model Calibration Result

The Response Surface Optimization feature in ANSYS, in conjunction with the collected testing data, was utilized to calibrate the model. The model was first parameterized with the inputs being the thermal conductivities of three materials with uncertain properties, stainless steel, glass, and HybridSil^®^, as well as the convection boundary condition. These input parameters were assigned a lower and upper bound based on reasonable expected values, as shown in Table 2.

For the outputs, various discrete locations and times were selected such that a direct comparison with the test data was possible. Using a Central Composite Design of Experiment, the parameterized model was solved 25 times, effectively covering the design space for each of the four input variables. Next, the relationship between the input variables and the output responses were quantified using full second-order polynomial response surfaces. The lowest coefficient of determination was 0.9997, indicative of a good fit of the response surfaces to the 25 generated design points.

Knowing the input–response relationships from the model, the Multiobjective Generic Algorithm (MOGA) was used to solve for the three unknown thermal conductivities and the convection boundary condition such that the response from the model matched the testing data, within reasonable uncertainty.

**Figure 5:**
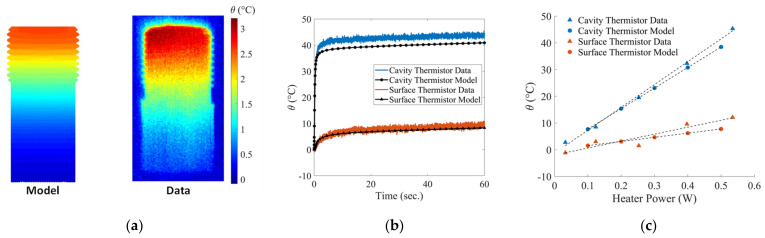
Detailed comparison between the experimental testing data and the calibrated model showing (**a**) the housing contour plots after 60 s, (**b**) cavity and surface thermistor temperatures over the initial 60 s period, and (**c**) cavity and surface temperatures for the range of heater power.

The model was calibrated with the testing data for the highest heater power (5 V), and a detailed comparison was performed to ensure the model was representative of the actual sensor. Figure 5 shows the comparison between the model and the test data, where θ is defined as the temporally local temperature minus the initial temperature.

The housing temperature contour plots in Figure 5a show that the model slightly underpredicts the temperature after 60 s; however, the model prediction is well within the uncertainty range of the IR camera. (±2 °C). Furthermore, the main interest is in the sensing element, so the slight difference in the housing temperatures is of little consequence.

The calibrated model’s prediction of the surface and cavity thermistor temperatures (Figure 5b) agrees well with the data in both trend and magnitude, with the largest difference of approximately 3 °C for the cavity thermistor. Lastly, the model and data were compared over the range of heater powers (Figure 5c) to ensure the model is valid for all heater powers, not just the 5 V case used for calibration. Comparing the slopes of the best fit lines, there is only an 8.6 °C/W and 10.4 °C/W difference for the cavity and surface thermistors, respectively, which is acceptable for the current purpose. 

The differences between the model and the data are possibly due to experimental uncertainty, imprecise selection of material properties, and the convection coefficient, during the calibration procedure, or the lack of a precise model for the thermistors. The calibrated model was taken to be representative of the actual sensor’s thermal performance and is now used for further development and investigation.

## 3. Results and Discussion

After the FE model was developed and calibrated via experimental testing data, the model was utilized to provide insight and understanding regarding the sensor’s temperatures, temperature distribution, potential sources of error during self-calibration, response time, and the efficiency of the thermopneumatic actuator. Two design studies were then conducted seeking to increase the efficiency of the sensor and to decrease the thermal response time of the actuator. The results, findings, and the potential implications for improving self-calibration performance are described in the following section.

### 3.1. Baseline Sensor

**Figure 6:**
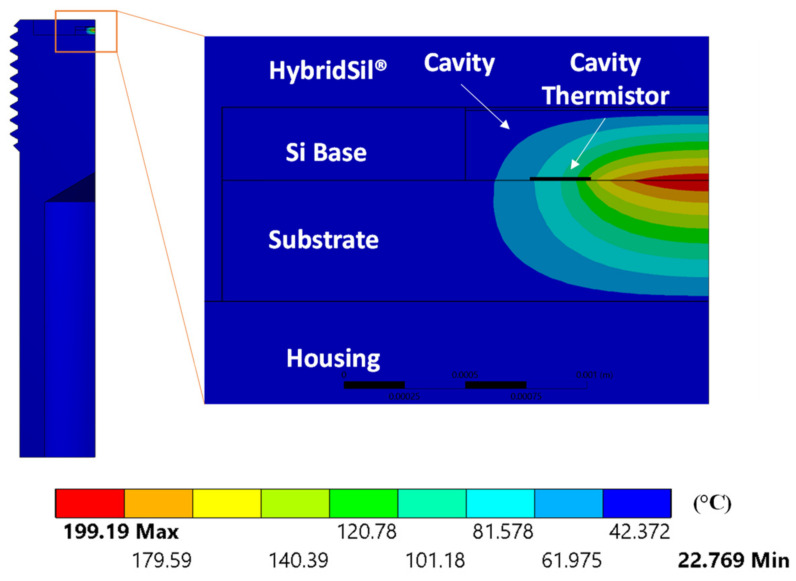
Temperature (°C) contour plot for the maximum heater voltage of 5 V.

The calibrated model was used to analyze and understand the thermal performance of the sensor, not observable during experimental testing. In particular, the temperature distribution in the sensing element and its effects were investigated. The temperature contour plot at the maximum heater voltage (5 V) is shown in Figure 6. As we were interested in the fastest possible response time (<30 s), the analysis hereafter is limited to the first 30 s.

A large temperature gradient was observed in the reference cavity with a maximum temperature of 199.2 °C at the bottom of the cavity where the heater was located and a minimum temperature of 29.4 °C. The temperature gradient was physical, as no convection (forced or buoyancy-induced) occurred in the cavity, as discussed previously. However, this temperature gradient was significant as the current self-calibration procedure assumed a uniform cavity temperature as measured by the cavity thermistor located at the bottom of the cavity, resulting in potential self-calibration error.

**Figure 7:**
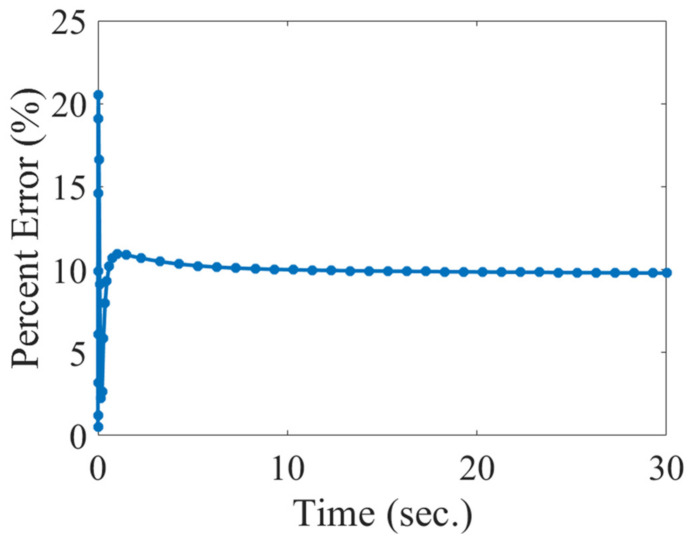
Estimated error between the average cavity temperature and the thermistor measured temperature.

When measuring the cavity bottom temperature, the thermistor will have an error associated with its measurement. To estimate this error, it was assumed that the actual reference cavity temperature was the volume average temperature in the cavity. The percent error between the cavity thermistor and the volume average temperature was calculated as a function time, as shown in Figure 7.

After about 10 s, the estimated error between the average cavity temperature and the thermistor measurement was approximately constant with a magnitude of 10%. This estimated error for the model is consistent with the approximate 6% error from previous experimental data [10]. The cavity thermistor over-predicts the cavity temperature and is attributed to the thermistor’s proximity to the heater. To achieve a more precise cavity temperature measurement, the location of the cavity thermistor should be moved away from the heater and ideally surrounded by the cavity gas only (suspended). 

A pressure sensor that can diagnose and correct measurement errors in real-time is valuable for time-sensitive applications such as flight. For this reason, the transient response of the self-calibration sensor was analyzed to minimize the time required to self-calibrate. The thermal time constant (τ), defined as the time required to change 63.2% from the initial to the final temperature, was used to determine how fast the actuator could respond thermally and, thus, how close to real-time self-calibration can be achieved. 

The temperature associated with 63.2% of change from the initial temperature to the temperature at 30 s was calculated, and linear interpolation was used to estimate τ between the discrete data points. The thermal time constant for the average cavity temperature and the cavity thermistor was 0.209 s and 0.264 s, respectively. The slight delay in the cavity thermistor response is attributed to the much lower thermal diffusivity of the glass substrate than air. The calculated thermal time constant for the baseline sensor is later used to evaluate various design options to minimize the thermal response time for the sensor.

**Figure 8:**
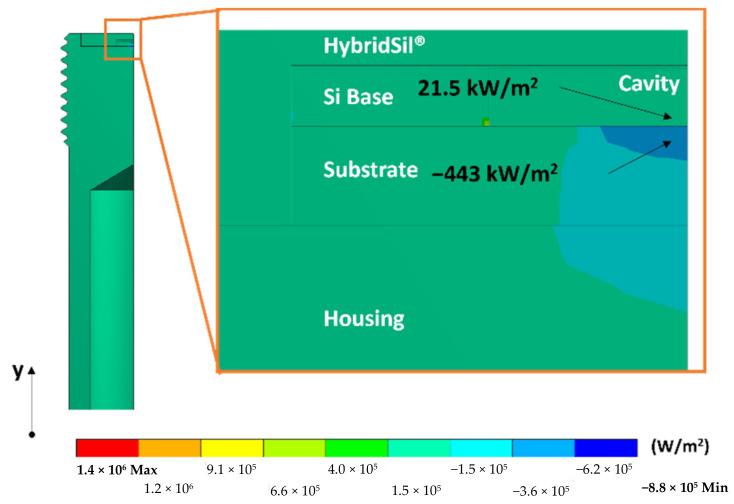
Heat flux contour plot in the vertical (y) direction.

Lastly, previous testing data indicated that more than 90% of heat is lost rather than used to heat the cavity. This heat loss reduces the energy efficiency of the sensor, critical for remote sensing applications. The developed model confirmed this inefficiency with the vertical direction heat flux contour plot in Figure 8.

Using point heat flux values from the contour plot, the model shows that 95.4% of heat was lost through the substrate. The heat loss is attributed to the low unit area thermal resistance of the substrate (4.48 × 10^−4^ m^2^ °C/W), compared to that of air in the cavity (8.58 × 10^−3^ m^2^ °C/W). The substrate is 94.3% less resistant to heat flow. This heat loss is significant and can be addressed by increasing the unit area thermal resistance of the substrate, as presented in the next section.

### 3.2. Substrate Material Selection

The observed heat loss was addressed by utilizing the model to determine potential design configurations resulting in higher efficiency. As previously mentioned, to increase the efficiency, the substrate’s thermal conductivity must decrease, increasing the unit area thermal resistance. Three alternative substrate materials were selected, focusing on materials with decreasing thermal conductivity: polyimide, a close-cell foam, and air.

Polyimide was selected for its good thermal and mechanical stability at high temperatures, its established use in electronics, and its proven manufacturing processes and techniques. Close-cell foam was selected for its high thermally insulating properties while providing a structure on which the heater could be manufactured. Lastly, the properties of air were used even though it is not possible to have an air substrate. This analysis was completed as a first approximation of a suspended heater, where air surrounds the microheater, as in MEMS gas sensors [20,21]. The suspended heater should provide higher efficiencies, as most of the heat would be transferred into the reference cavity gas, rather than being conducted away through the surrounding structures. The selected materials and their properties are listed in Table 3.

To analyze the effect of the substrate material on the actuator’s efficiency, the average cavity temperature results were plotted over the full range of heater powers, and the data were fitted with linear trend lines. Comparing the slopes (a) of the trend lines, it is observed that for decreasing thermal conductivity, higher cavity temperatures are achieved per watt of heater power. By changing from the current glass substrate to a polyimide, foam, or air (suspended heater) substrate, the power consumption can be lowered by 81.5%, 85.2%, or 92%, respectively. The authors attribute this to the increasing unit area thermal resistance of the substrate, redirecting heat up into the cavity. The results are summarized in Table 3.

The effect of changing the substrate material on the transient response was also considered. The transient response is evaluated by calculating the thermal time constant (τ) from the temporal temperature data. The results show that a lower thermal conductivity, required for less heat loss, does not always result in a faster response. Instead, a substrate material with high thermal diffusivity (α) exhibits a faster response time. Thus, according to Equation (5), for a material with low thermal conductivity to have a high thermal diffusivity, the density and specific heat need to be small.

The results from the substate material study are summarized in Table 3 and show that optimal efficiency and fast transient response can be achieved by utilizing a substrate with low thermal conductivity and high thermal diffusivity.

### 3.3. Cavity Size

A smaller cavity is of interest, as the amount of air to be heated is decreased, potentially increasing efficiency, and reducing the actuator’s response time. Using the previously calibrated numerical model, a parametric design study was conducted to predict the effects of altering cavity dimensions on self-calibration.

**Figure 9:**
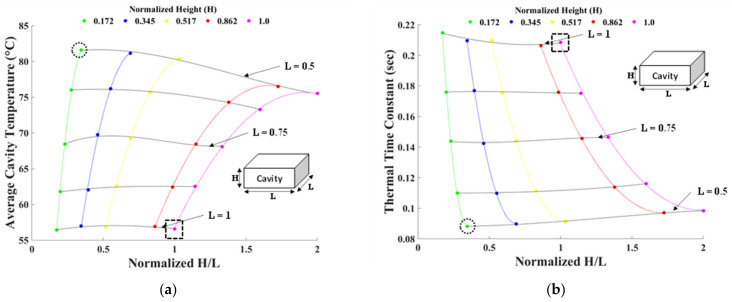
Cavity size design study results showing (**a**) the average cavity temperature and (**b**) the thermal time constant for decreasing cavity base dimension and cavity height.

The heater power was held constant at 0.5 W while varying both the square cavity base dimension (L = 2 mm) and the cavity height (H = 0.29 mm). Starting from the current sensor configuration, L and H were incrementally reduced independently and simultaneously. As in the substrate material study, the effects of the cavity size on the efficiency and response time were evaluated. The cavity base and height dimensions were normalized using the dimensions of the baseline sensor. The resulting average cavity temperature (Figure 9a) and the thermal time constant (Figure 9b) results were plotted against a ratio of the normalized height to the normalized base dimensions. The colored lines in the vertical direction indicate a constant cavity height (H), and the black lines in the horizontal direction indicate a constant cavity base dimension (L).

The results generally indicate that cavities with smaller volumes (smallest volume investigated marked by a dashed circle in Figure 9) achieve faster response than larger cavities (largest cavity volume investigated marked by a dashed square in Figure 9). The trends in Figure 9 also show the sensitivity for changing the cavity base versus changing the cavity height. Starting from the current sensor’s cavity size, indicated by the dashed squares, the average cavity temperature and the thermal time constant are substantially more sensitive to a decrease in the base dimension than the height. A 50% decrease in the base dimension is predicted to result in a 45% increase in temperature and a 59% decrease in the thermal time constant, whereas an 83% decrease in cavity height leads to a marginal predicted temperature increase of only 8% and a 10% decrease in the thermal time constant. The higher sensitivity on the cavity base dimension is attributed to the L^3^ term when calculating the volume average temperature, on which the thermal time constant is also dependent. This parabolic nature of the constant–height (altering base dimension) lines indicates an optimal point at which a maximum temperature and a minimal thermal time constant will be achieved for a given cavity height and heater power. Reducing the base cavity dimension any further will have minimal adverse effects on the heating efficiency and response times. 

The results from this cavity size study confirm that a sensor with a smaller cavity will be more efficient and actuate faster. Moreover, the study shows when decreasing the cavity size that efforts should be focused on reducing the cavity base dimension first.

### 3.4. Implications for Sensor Improvements

Based on the results of the numerical study, it is found that one of the limiting factors for the current sensor design performance is heat loss. The simulation with the air being the substrate indicates that a suspended heater structure results in significantly better insulation, thus enhancing the power efficiency, which agrees with the results in [20,21]. Another limiting factor is found to be the cavity size. Reducing the base dimension of the sensor cavity is proven to decrease the response time to reach the required thermal status. A faster response time and a lower heat power demand are critical to the sensor performance. These improvements will be considered in the team’s future design of the self-calibration sensor.

New fabrication challenges will arise as these improvements are implemented. For example, supporting a suspended heater in this microscale cavity can cause sealing and structural integrity problems. Meanwhile, reducing the cavity size sets a higher standard of micromachining. In order to tackle these challenges, further prototype manufacturing and experimental tests are needed and will be shown in the team’s future work.

## 4. Conclusions and Future Work

The potential uses and benefits of a thermopneumatic actuated self-calibrating pressure sensor are significant. However, predictive and consistent thermal performance is required for accurate self-calibration. The present work used a finite element model calibrated with experimental data to increase understanding of the thermal performance of the sensor. The model shows that a temperature gradient in the reference cavity resulted in an error estimated to be 10%, as a point measurement is taken as the bulk cavity temperature. The model also confirmed that most heat is lost, as approximately 95.4% of heat transfers through the substrate due to its lower thermal resistance than air. Using the model as a design tool, two potential design improvements were thoroughly analyzed to increase efficiency and minimize the thermal response time. The substrate material design study showed that optimal efficiency, high temperature, and a fast response can be achieved with a substrate with low thermal conductivity and high thermal diffusivity. Lastly, it was found that the cavity base dimension should be decreased to reduce response time. A third design study was conducted to study the effects of the reference cavity gas on the sensor’s responses. However, the study revealed insignificant changes. The reference cavity gas study can be found in [18].

The presented work increased understanding and provided insight into potential design improvements for the sensor by Kang et al., moving one step closer to optimized self-calibrating pressure sensors. Future work on the presented model should focus on installing a suspended heater and reducing the base size of the sensing cavity. The team will also work on material property refinement and increasing the model’s complexity by modeling the joule heater, thermistors, and the effects of nonperfect thermal contact between parts. Lastly, a multiphysics (thermal, fluid, and structural) model can be developed to accurately predict cavity pressures and account for effects such as thermal-induced stress and the fluid and structural damping effects on the transient response of the sensor.

## Figures and Tables

**Table 1:** Initial thermal diffusivity of the sensor materials.

Component	Material	Thermal Diffusivity·10^−6^ (m^2^/s)
Base layer	Silicon (Si)	89.21
Buried oxide (BOX) layer	Silicon dioxide (SiO_2_)	0.83
Cavity	Air (400 K)	38.27
Substrate	Glass	0.65
Housing	Stainless steel	4.05
Protective coating	HybridSil^®^	0.11

**Table 2:** Lower and upper bounds for the model calibration parameters.

Parameter	Lower Bound	Upper Bound
k_glass_ (W/m·°C)	0.8	0.5
k_stainless_ (W/m·°C)	12.5	17.5
k_HybridSil_ (W/m·°C)	0.1	0.5
h (W/m^2^·°C)	1	200

**Table 3:** Material properties and results from the substrate material design study.

Material	k (W/m^2^ °C)	α·10^6^ (m^2^/s)	a (°C/W)	Power Consumption Decrease (%)	τ (s)
Glass (baseline)	1.034	0.559	63.38	N/A	0.209
Polyimide	0.12	0.078	341.93	81.46	0.706
Foam	0.085	1.504	428.89	85.22	0.034
Air	0.034	38.265	792.32	92.00	0.002

## Data Availability

The data presented in this study are available on request from the corresponding authors.

## Nomenclature

*R*	Electrical resistance
π	Piezoresistive coefficient
σ	Stress
*P*	Pressure
*Gr*	Grashof number
*g*	Gravitational acceleration constant
β	Volumetric thermal expansion coefficient
*T*	Temperature
*L*	Length
ν	Kinematic viscosity
ρ	Density
*C_p_*	Specific heat
*t*	Time
*U*	Velocity
*k*	Thermal conductivity
*q‴*	Volumetric heat generation
α	Thermal diffusivity
*h*	Convection coefficient
θ	Initial temperature compensated temperature
τ	Thermal time constant

## References

[1] Kumar S.S., Pant B.D. (2014). Design Principles and Considerations for the “ideal” Silicon Piezoresistive Pressure Sensor: A Focused Review. Microsyst. Technol..

[2] Tran A.V., Zhang X., Zhu B. (2018). Mechanical Structural Design of a Piezoresistive Pressure Sensor for Low-Pressure Measurement: A Computational Analysis by Increases in the Sensor Sensitivity. Sensors.

[3] Aryafar M., Hamedi M., Ganjeh M.M. (2015). A Novel Temperature Compensated Piezoresistive Pressure Sensor. Measurement.

[4] Chou T.L., Chu C.-H., Lin C.-T., Chiang K.N. (2009). Sensitivity Analysis of Packaging Effect of Silicon-Based Piezoresistive Pressure Sensor. Sens. Actuators A Phys..

[5] Campabadal F., Carreras J.L., Cabruja E. (2006). Flip-Chip Packaging of Piezoresistive Pressure Sensors. Sens. Actuators A Phys..

[6] Bae B., Flachsbart B.R., Park K., Shannon M.A. (2004). Design Optimization of a Piezoresistive Pressure Sensor Considering the Output Signal-to-Noise Ratio. J. Micromech. Microeng..

[7] Meijer G., Pertijs M., Makinwa K. (2014). Smart Sensor Systems: Emerging Technologies and Applications.

[8] Yameogo P., Heiba U., Al Bahri M., Pons P. Self Calibrating Pressure Sensor for Biomedical Applications. Proceedings of the IEEE Sensors 2009.

[9] Puers R., Reyntjens S., De Bruyker D. (2001). Remote Sensors with Self-Test: New Opportunities to Improve the Performance of Physical Transducers. Adv. Eng. Mater..

[10] Kang Y., Sibold R., De Clerck A., Ng W., Ruan H. Self-Calibrating and Conformal Pressure Sensors for Embedding Sensing Applications. Proceedings of the ASME Turbo Expo 2020.

[11] Oh K.W., Ahn C.H. (2006). A Review of Microvalves. J. Micromech. Microeng..

[12] Laser D.J., Santiago J.G. (2004). A Review of Micropumps. J. Micromech. Microeng..

[13] Puers R., De Bruyker D., Cozma A. (1997). A Novel Combined Redundant Pressure Sensor with Self-Test Function. Sens. Actuators A.

[14] De Bruyker D., Cozma A., Puers R. (1998). A Combined Piezoresistive/Capacitive Pressure Sensor with Self-Test Function Based on Thermal Actuation. Sens. Actuators A Phys..

[15] Ozsun O., Alaca B.E., Yalcinkaya A.D., Yilmaz M., Zervas M., Leblebici Y. (2009). On Heat Transfer at Microscale with Implications for Microactuator Design. J. Micromech. Microeng..

[16] Damean N., Regtien P.P.L., Elwenspoek M. (2003). Heat Transfer in a MEMS for Microfluidics. Sens. Actuators A Phys..

[17] Guo Z.-Y., Li Z.-X. (2003). Size Effect on Microscale Single-Phase Flow and Heat Transfer. Int. J. Heat Mass Tran..

[18] De Clerck A. (2020). Modeling the Thermal Performance of an Intelligent MEMS Pressure Sensor with Self-Calibration Capabilities. Master’s Thesis.

[19] Cole K.D., Tarawneh C., Wilson B. (2009). Analysis of Flux-Base Fins for Estimation of Heat Transfer Coefficient. Int. J. Heat Mass Tran..

[20] Simon I., Bârsan N., Bauer M., Weimar U. (2001). Micromachined Metal Oxide Gas Sensors: Opportunities to Improve Sensor Performance. Sens. Actuators B Chem..

[21] Bhattacharyya P. (2014). Technological Journey towards Reliable Microheater Development for MEMS Gas Sensors: A Review. IEEE Trans. Device Mater. Reliab..

